# Preparing the AI-Ready Dentist: A Call for a Competency Framework in Dental Education

**DOI:** 10.1016/j.identj.2026.109450

**Published:** 2026-02-19

**Authors:** Thanaphum Osathanon, Anjalee Vacharaksa, Falk Schwendicke, Lakshman Samaranayake

**Affiliations:** a Dental Education Unit, Office of Academic Affairs, Faculty of Dentistry, Chulalongkorn University, Bangkok Thailand; b Center of Artificial Intelligence and Innovation, Faculty of Dentistry, Chulalongkorn University, Bangkok Thailand; c Center of Excellence for Dental Stem Cell Biology, Department of Anatomy, Faculty of Dentistry, Chulalongkorn University, Bangkok Thailand; d Master of Science Program in Geriatric Dentistry and Special Patients Care, Faculty of Dentistry, Chulalongkorn University, Bangkok Thailand; e Research Unit on Oral Microbiology and Immunology and Department of Microbiology, Faculty of Dentistry, Chulalongkorn University, Bangkok Thailand; f Conservative Dentistry, Periodontology and Digital Dentistry, LMU Hospital, Munich Germany; g Faculty of Dentistry, The University of Hong Kong, Special Administrative Region, Hong Kong China; h Global Research Cell, Dr. D. Y. Patil Dental College and Hospital, Dr. D. Y. Patil Vidyapeeth, Pune India

**Keywords:** Artificial intelligence, Curriculum, Dental education, Competency

## Abstract

Rapid advances in artificial intelligence (AI) that assist clinical workflows and processes demand systematic educational strategies to cultivate AI competencies within dental curricula. This perspective calls for prioritising educational initiatives to create an AI-proficient dental workforce. AI education should be integrated vertically throughout preclinical to clinical years while ensuring horizontal coherence with existing competencies. Early-stage students should focus on beginner-level competencies, acquiring foundational AI knowledge and ethical considerations. Senior learners should demonstrate the ability to implement AI tools for clinical tasks and to critically interpret AI-generated outputs. Advanced students should be equipped with skills to innovate AI-driven studies and novel applications for oral healthcare. Assessments should also be well designed to capture and evaluate the expected AI competencies. As curricula include massive amounts of both technical and nontechnical content, integrating AI teaching and learning must be carefully balanced with the core competencies required of dental professionals.

## Introduction

The introduction of Artificial Intelligence (AI) has facilitated digital transformation in all healthcare sectors. In dentistry, AI-based tools and applications are increasingly utilised as adjuncts to improve the efficacy, safety, consistency, and efficiency of clinical procedures, ranging from data collection and diagnostic assistance to decision-making and beyond.^1^^,^^2^ The inherent dynamic nature of AI technology, however, poses challenges for educators preparing a new generation of dental professionals for the era of AI. Hence, the curriculum of both predoctoral and postdoctoral dental programs must equip students to adapt to the rapidly developing technologies and to utilise them proficiently.

Multiple institutions across different geographic regions have incorporated components of AI-related competencies into their curricula to varying extents. The specific content and learning objectives often differ based on local contextual factors, existing expertise and infrastructure, and the information available. The emergence of commercially available novel AI tools in dental practices, together with the rapid advancement of large language models, highlights the urgent need for a standardized framework of competencies and strategic approaches to integrate AI knowledge and skills into dental education.

**Fig. 1:**
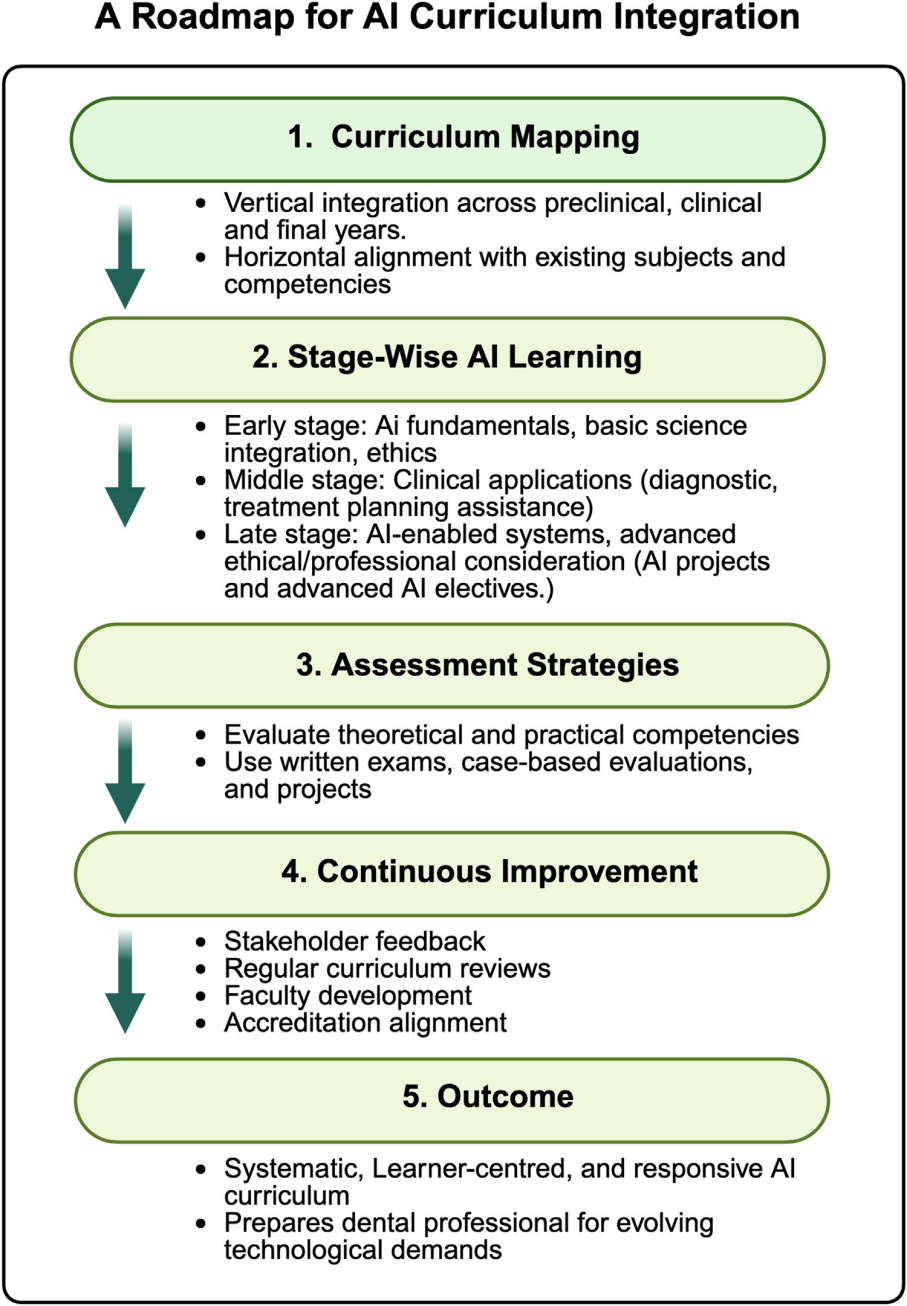
A proposed staged pathway for integration of AI into the dental curriculum roadmap. Created in BioRender. https://BioRender.com/9ls82k8.

Implementation of the proposed AI curriculum framework requires a structured approach encompassing curriculum mapping, assessment strategies, and continuous improvement (Figure 1).^3^ In this perspective, a generic AI framework for the dental curriculum is proposed and highlighted for implementation. The alignment and integration of AI competencies, both vertically and horizontally, are discussed. The proposed framework, combined with the WHO/ITU core curriculum, offers a robust pathway for integrating AI into dental curricula. This perspective calls on all stakeholders to prioritise this educational imperative by leveraging existing data to develop an AI-literate dental workforce that is equipped to meet future demands.

## The imperative for a structured framework for AI literacy

Integrating AI into the dental curriculum poses substantial challenges, including content analysis of required AI competencies, vertical integration of AI competencies into the curriculum, horizontal integration of AI with core competencies of other subjects, and the identification of skills needed to address the rapid evolution of AI knowledge after graduation. To address these challenges, it is essential to develop standardised protocols that define the key components of AI literacy and provide guidance on effective instructional methods.^4^

Presently, curriculum organisers often adopt a reactive approach, for example, by introducing isolated software tools without sufficient context and formal AI education. To mitigate this gap, AI instruction should precede or accompany practical application. Teaching the foundational principles, the “why” and “how,” is as essential as explaining the “what” and “where” of its use. Targeted educational initiatives should be designed to provide specialised training in AI concepts, ethical considerations, and practical applications while promoting critical thinking to ensure learners understand the limitations, biases, and broader implications of AI technologies.^5^

However, these components are frequently provided in a fragmented manner, limiting their translation into cohesive, tangible learning outcomes. Global bodies echo the call for structured AI education. The FDI World Dental Federation has emphasised that oral health professionals must be equipped to navigate the digital transformation,^6^ and both FDI and the Australian Dental Association released policy statements on AI in Dentistry, highlighting the need for structured education on AI.^7^ UNESCO has provided overarching guidance on developing AI competencies for both students and teachers, stressing ethics and human oversight.^8^^,^^9^ A possible core curriculum and required outcome regarding oral and dental AI have been defined by the ITU/WHO Focus Group AI for Health in collaboration with IADR’s e-oral health group and the Association for Dental Education in Europe.^10^

## The core domains: building knowledge, skill, and wisdom

In this context, 4 primary domains have been defined,^10^ that outlines key areas that dental professionals should be aware of, namely, (i) overview of AI and their uses in medical applications, (ii) AI in oral and dental healthcare and the potential applications, (iii) assessing procedures for medical and dental AI, and (iv) broader considerations relevant for dental professionals when engaging with AI technologies.

The curriculum should focus on these and ensure that the students achieve the “show how” or “does” levels based on Miller’s Pyramid of Competence.^11^ Based on the aforementioned study on the core educational curriculum for oral and dental AI, the 4 interconnected aspects have been further defined aiming to develop not only technical proficiency but also critical judgment and ethical responsibility in the application of AI within dental practice.

1. *AI fundamentals*—*cultivating understanding.* Aligned with the first domain mentioned above, this foundational layer moves students from awareness to comprehension. It involves grasping basic concepts of all aspects of AI in dentistry and developing the skill to discover and ethically experiment with emerging tools. The goal is to elucidate the complexities of AI, replacing apprehension with informed curiosity. In this respect, the “know-how” levels could be set as the standard learning outcome.
2. *AI implementation*—*developing application*. In addition to the knowledge levels described in the second domain above, competencies include critically applying AI tools to specific tasks and understanding the processes of designing, training, and validating dental AI models. In this regard, students should attain the “does” levels to effectively demonstrate their ability to utilize or innovate AI appropriately for oral and dental applications, ensuring compliance with both technical and ethical standards.
3. *Integrating critical thinking with AI—fostering judgment*. Integrating critical thinking into AI education is not optional; it is imperative. Insufficient application of critical thinking heightens the risk of AI misuse, while excessive reliance on AI may compromise clinical judgment and decision-making.^12^ AI can also support the development of critical thinking outcomes, complemented by structured learning guides and robust assessment strategies.^13^

The learning experiences that foster critical thinking about AI aspects must be integrated vertically as well as horizontally across the curriculum. Corresponding with domains (iii) and (iv) above, students must be equipped with the knowledge and skills in metrics, interpretation, and impact on health outcomes.

**Fig. 2:**
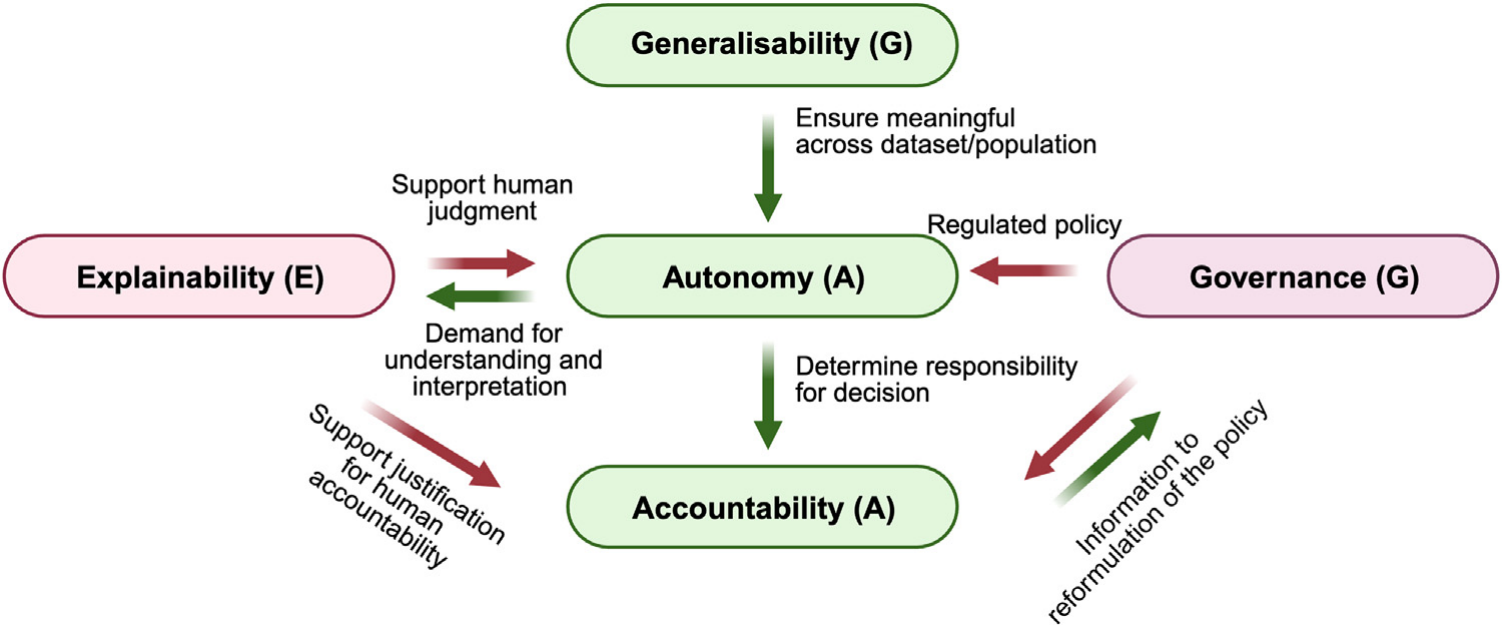
A conceptual layout diagram that visually connects the 5 core components of artificial intelligence systems in dentistry (acronym: GEAGA framework).

Understanding the concept of AI utilisation in dentistry is also essential for judgment, including generalisability (G), explainability (E), autonomy (A), governance (G), and accountability (A).^1^^,^^14^ An AI system should perform reliably across diverse populations and data sources, reflecting its generalisability. Explainability is a crucial parameter that enables transparency of algorithms to be interpreted and validated by the involved parties. Students must also recognise the autonomy of AI use. AI functions as an assistive tool; hence, the judgment remains a human responsibility. In addition, the school and university should provide an AI governance policy to guide students in relation to the institution’s standard regulations. Lastly, students must realise that they are fully accountable for the decision and the outcome after utilising AI. A unified conceptual framework incorporating the 5 core pillars above, which we propose as the GEAGA concept (Figure 2), offers a novel lens for evaluating and guiding the effective integration of AI technologies in dental education settings.

With this fundamental information, students can develop critical thinking skills in AI, enabling them to evaluate, identify, and mitigate the benefits and risks of AI applications in oral and dental health. Learning experiences such as problem-based and case-based learning can provide opportunities to cultivate these skills and to evaluate the “show-how” levels as students demonstrate them in a real-world environment.

*4. Ethical and professional conduct*—*ensuring stewardship.* This focuses on the responsible use of AI, ensuring patient data privacy and informed consent. It reinforces the nonnegotiable role of human oversight in clinical decision-making. This domain prepares graduates to be ethical stewards of technology in their communities. The “know-how” levels should be set as the learning outcome levels in this perspective.

Although the core domains of content and learning experiences are clearly defined, the assessment procedure must also be structured to align with the intended learning outcome levels, ensuring that the students’ abilities are accurately reflected and assessed. A traditional examination can be employed to evaluate fundamental knowledge at the “know” and “know-how” levels. However, for the higher learning outcome levels (“show how” and “does”), it is essential to construct appropriate rubric criteria for assessment, whether in a set environment or in authentic clinical tasks, to capture students’ performance accurately.

AI competencies should be assessed at the higher levels of Miller’s Pyramid to capture learners’ ability to integrate AI into authentic clinical reasoning. At the “shows how” level, case‑based simulations can incorporate AI tools, such as an objective structured clinical examination (OSCE) station in which a digital platform presents intraoral images alongside AI‑generated differential diagnoses for conditions like lichen planus, leukoplakia, and early oral squamous cell carcinoma. These assessments evaluate learners’ capacity to critically appraise AI outputs, apply them appropriately within their diagnostic reasoning, and communicate decisions while maintaining patient safety. Another example is that an OSCE station incorporating AI‑assisted simulation in caries‑risk assessment and clinical decision‑making can allow students to “show how” they safely interpret an AI caries‑risk tool, communicate its findings to a simulated adult patient, and engage in ethical shared decision‑making. This structured practice in a controlled environment builds confidence and prepares students for assessment at the “does” level in real clinical settings. At the “does” level, workplace‑based assessments further examine how clinicians use AI responsibly in real practice, with emphasis on ethical awareness, recognition of algorithmic bias, and avoidance of overreliance on automated suggestions. This approach ensures that AI‑related competencies reflect not only technical proficiency but also sound clinical judgment and professional responsibility.

### Crafting the framework: a synthesis of vision and practice

Developing a robust competency framework requires aligning theoretical foundations with clinical practice. The WHO/ITU AI Core Curriculum for dental education followed the step-wise processes listed below:^10^

• Reviewing the existing curricula,
• Conducting expert interviews,
• Defining learning outcomes,
• Structuring the domains, and
• Applying a Delphi process.

The development of an AI curriculum framework should begin by establishing goals informed by stakeholder requirements, including input from a multidisciplinary expert panel and alignment with institutional strategies and guiding principles. The framework should emphasise integrating AI applications across all relevant domains, structured through a staged process within a learner-centered design.

The learning outcomes should ensure that learners develop both a solid theoretical foundation and practical competencies for applying AI across diverse clinical contexts. A learner-centered design promotes engagement and adaptability, preparing graduates to meet the evolving technological demands of healthcare. Additionally, strict adherence to accreditation standards is essential to ensure educational quality, uphold institutional credibility, and maintain compliance with regulatory and professional requirements. Collectively, these principles provide a robust foundation for developing an AI curriculum that is academically rigorous, clinically relevant, and contextually relevant.

The proposed AI competency framework aligns proficiency levels with the progressive nature of dental education. Undergraduate programs should focus on beginner-level competencies, ensuring students develop foundational knowledge of AI principles, terminology, and ethical considerations. At this early stage, junior students should be able to define key concepts, recognise basic applications in dentistry, and identify potential ethical and regulatory issues.

As students advance into clinical years and postgraduate training, the emphasis shifts to intermediate and advanced competencies. Senior learners should demonstrate the ability to apply AI tools in diagnostic and treatment planning, critically interpret AI-generated outputs, and integrate these insights into complex clinical decision-making. Furthermore, postgraduate education should cultivate advanced research skills, enabling learners to design and execute AI-driven studies and innovate new applications for oral healthcare.

Across all levels, adherence to professional and ethical standards remains essential. While undergraduates should understand the principles of fairness and transparency, postgraduate learners must be prepared to implement compliance measures and contribute to institutional policy development. This staged approach ensures that graduates are not only AI-literate but also capable of leveraging AI responsibly and effectively across diverse clinical and research contexts.^10^^,^^15^

## A roadmap for curriculum integration

Curriculum mapping should integrate AI education vertically across preclinical, clinical, and final years while ensuring horizontal alignment with existing subjects and competencies. Early stages introduce AI fundamentals alongside basic sciences and ethics, progressing to clinical applications in diagnostic^16^ and treatment planning,^17^ AI-enabled virtual reality systems for dental education,^18^ and culminating in advanced ethical and professional considerations through projects and electives. Assessment strategies must evaluate both theoretical knowledge and practical competencies, employing written examinations, case-based evaluations, and projects to ensure learners can critically appraise and apply AI tools.^19^ A continuous improvement process, supported by stakeholder feedback, regular curriculum reviews, faculty development, and adherence to accreditation standards, is essential to maintain relevance and quality. Collectively, this approach ensures that AI integration is systematic, learner-centered, and responsive to evolving technological and professional demands.

The implementation of an AI curriculum framework may be challenging in settings with limited resources and infrastructure. In such contexts, the framework can prioritize AI literacy and ethical practice as core competencies, even in the absence of advanced or up‑to‑date equipment. AI‑related skills can be adapted to available resources while emphasizing self‑directed learning as a foundation for lifelong learning. This flexible approach enables students to achieve the intended learning outcomes and enhances the global applicability of the proposed framework.

Faculty development is a critical component for the success of implementation. It requires a structured and progressive approach. At the foundational level, all faculty members should receive training in AI literacy, ethical principles, and responsible use, ensuring consistent integration of these competencies across educational activities. In the next phase, faculty with interest or discipline‑specific needs may pursue more advanced training focused on the pedagogical integration of AI tools for teaching, assessment, and clinical decision support. These opportunities should be available for faculty seeking specialized expertise, including AI‑enhanced research, curriculum innovation, or leadership roles in educational transformation. To sustain this progression, institutional policies, incentives, and infrastructural support are essential to promote faculty engagement, recognize competence development, and allocate protected time for training. Through this tiered development model, faculties are equipped to function both as competent providers of AI‑informed education and as facilitators of student‑centered, lifelong learning experiences.

## Conclusion: leading the digital transformation

The journey to integrate AI into dental education is not about chasing the latest technological trend. It is a fundamental responsibility to prepare graduates for the profession they will inherit, i.e., one where digital and clinical intelligence are inextricably linked. A competency framework spanning the curriculum provides the essential map for this journey. Today, the question is not “whether” AI must be taught in the dental curriculum, but “how” it should be taught systematically, ethically, and effectively. The systematic implementation of AI competencies in the dental curriculum, along with the practical assessment of learning outcomes at expected levels, ensures that graduates can harness AI to elevate the standard of patient care and shape a more equitable future for oral health. Given the already intensive content and tightly structured schedule encompassing both technical and nontechnical skills in dentistry, integrating teaching and learning experiences related to AI competencies into the dental curriculum presents a significant challenge. Such integration must be carefully balanced with the existing core competencies required of dental professionals.

Moreover, cross-disciplinary and interprofessional education is being increasingly integrated into dental training curricula to strengthen holistic care coordination and patient safety. AI‑assisted learning can provide practical, team‑based scenarios for interprofessional education, including shared case‑based seminars with medicine, nursing, and pharmacy programs; joint simulations using AI‑based decision support; and cosupervised quality improvement projects. These activities are feasible even in resource‑constrained settings. Interprofessional practice is particularly important for vulnerable populations, such as older adults with complex needs, including polypharmacy, frailty, and dementia, as well as patients requiring special care, such as individuals with neurodevelopmental disorders or medically complex conditions. These groups often present multifaceted health challenges that necessitate coordinated input from a multidisciplinary team. Lastly, we acknowledge that AI technologies evolve more quickly than traditional educational content; therefore, adopting an evergreen curriculum structure with scheduled updates, modular learning units, and ongoing horizon scanning processes can ensure sustained curricular relevance.

## Author contribution

TO and LS contributed to the conceptualization and drafted the original manuscript. AV and FS contributed to the critically edited manuscript. All authors approved the manuscript for submission.

## Declaration of generative AI and AI-assisted technologies in the writing process

During the preparation of this work, the authors used AI tools (ChatGPT and SciSpace) to summarise the AI Framework document in the Faculty of Dentistry, Chulalongkorn University, and to translate the drafts (Thai) into English. AI tools (ChatGPT and Grammarly) were also used to improve its readability and language. After using this tool/service, the authors reviewed and edited the content as needed and take full responsibility for the publication's content.

## Conflict of interest

None disclosed.
